# Screening of Mycotoxigenic Fungi in Barley and Barley Malt (*Hordeum vulgare* L.) Using Real-Time PCR—A Comparison between Molecular Diagnostic and Culture Technique

**DOI:** 10.3390/foods11081149

**Published:** 2022-04-15

**Authors:** Marina Bretträger, Thomas Becker, Martina Gastl

**Affiliations:** Chair of Brewing and Beverage Technology, TUM School of Life Sciences, Technical University of Munich, 85354 Freising, Germany; marina.brettraeger@tum.de (M.B.); tb@tum.de (T.B.)

**Keywords:** black fungi, contamination, Dematiaceae, food safety, *Hordeum vulgare*, malting barley, mycotoxins, real-time PCR

## Abstract

Filamentous fungi have a crucial impact on the food safety and technological quality of malting barley. Commonly used techniques for the detection of seed-borne fungi are based on cultivation and identification by morphological criteria. In contrast, this study established a quantitative real-time polymerase chain reaction (PCR) assay based on SYBR green technology for the detection and quantification of black fungal species (*Alternaria* spp., *Epicoccum nigrum*, *Cladosporium cladosporioides*, *Penicillium verrucosum* and *Aspergillus niger*) on brewing barley and compares it with the traditional cultivation technique and visual assessment. To screen the fungal spectrum over different barley varieties and harvest years, naturally infected samples of malting barley and corresponding malts (*Hordeum vulgare* L.) were analyzed over four consecutive years (2018–2021), grown under different climatic conditions in Germany. *Alternaria* and *Cladosporium* spp. DNA were present in all examined barley samples, even without visible contamination. In contrast, detection via culture-based methods does not reliably cover all species. Molecular analysis showed that there was less fungal biomass after malting, by 58.57% in the case of *A. alternata*, by 28.27% for *Cladosporium* spp. and by 12.79% for *Epicoccum nigrum*. Correlation analysis showed no causal relationship between fungal DNA and the number of black kernels. The qPCR provides a highly sensitive and time-saving screening method for detecting latent fungal infections in brewing grains to identify batches that are potentially highly contaminated with toxigenic fungi.

## 1. Introduction

Barley (*Hordeum vulgare* L.) is the fourth most cultivated cereal crop worldwide, used predominately in animal feed, human consumption and, with specialized qualitative requirements, in the malting and brewing industry. The majority of spring barley grown in Germany is produced for malt production. In 2020, the cultivation area for spring barley in Germany was approximately 353,000 ha, with a crop volume of 1.904 million tons [1]. The grains are naturally exposed to many microbial infections and contamination in their entire life cycle. Ubiquitous fungi infect barley plants in the field or spoil the harvested barley and malt during storage and processing. In areas that rely on flawless raw materials, such as malt houses and breweries, the presence of filamentous fungi is a major concern.

From a technological viewpoint, fungal persistence negatively affects storage quality and processability. It is known from the literature that it reduces germination capacity, as secreted fungal hydrolytic enzymes alter malt modifications and lead to variable malt quality, consequently influencing malting and brewing performance and the properties of the final beer [2,3,4,5]. Apart from technological problems related to the handling of the contaminated cereals, processing is also connected with potential human health risks for consumers, in terms of the threshold of toxicological concern (TTC) value for mycotoxins. Various fungal species are capable of producing a wide range of resilient toxic secondary metabolites, called mycotoxins, which are harmful to health. The tolerable daily intakes for certain mycotoxins are set by the European Food Safety Authority (EFSA).

In terms of quality control, mycotoxins are the driving force, as they are potentially transferable from grains to malt and may be released during brewing into the final product, beer, depending on their solubility [6,7,8,9,10,11,12].

Extreme weather conditions caused by global climate change are leading to an increased incidence of fungal infestation. Therefore, mycotoxins are increasingly coming into focus, especially in temperate climates, as rising temperatures have also had a significant impact on the growth of mycotoxigenic microorganisms and the formation of toxins [13,14]. 

The process of malting is the controlled germination of cereal grains; its main purpose is the activation and new formation of enzymes to dissolve the endosperm [15]. The temperatures and high moisture conditions during the malting process (steeping, germination, kilning) favor the growth of filamentous fungi. Therefore, there is a risk that the mycotoxin concentration in malt can be drastically increased compared to barley, depending on the fungus [8,11]. 

Mycotoxins are very stable compounds whose formation can best be prevented by controlling fungal growth during storage and malting. However, if the initial contamination level is already very high in the raw material (barley), these are of limited use because the focus must be on processing to maintain malt quality. Therefore, it is important to detect latent fungal infections early enough in order to identify batches that are potentially highly contaminated with toxigenic fungi. 

Several works have summarized the mycoflora of malting barley using traditional cultivation techniques [16,17,18]. The main representatives found on European malting barley belong to the genera *Fusarium*, *Alternaria*, *Cladosporium*, *Penicillium*, *Aspergillus*, *Drechslera* (*Helminthosporium*), *Mucor* and *Epicoccum*. Aflatoxins (AFs), ochratoxin A (OTA), trichothecenes, fumonisins (FBs), zearalenone (ZEA) and citrinin are the most frequently detected mycotoxins in malting barley [17,19,20,21]. In recent studies, *Alternaria* mycotoxins have been coming into focus [22,23]. The most frequent *Alternaria* toxins are alternariol (AOH), alternariol monomethyl ether (AME), altenuene (ALT), tenuazonic acid (TeA) and altertoxins (ATX), tentoxin (TEN) and some modified forms. OTA is often produced by *Aspergillus* and *Penicillium* species. *P. verrucosum* is the major producer of ochratoxin A (OTA) in temperate climates. In addition to OTA, it has also been reported that *P. verrucosum* forms the mycotoxin citrinin. *A. niger* is known to produce two groups of potentially carcinogenic mycotoxins: ochratoxins and fumonisins, mainly fumonisin B2. Members of the genus *Cladosporium* have a ubiquitous distribution; they are found in soil, air and plant debris. Salvatore et al. [24] has reviewed bioactive secondary metabolites produced by the species of *Cladosporium*, including some substances with cytotoxic and toxic properties, such as cladosporin or fusarubin [25]. Currently, there is no known information on the toxicity of *Epicoccum nigrum* (syn. *Epicoccum purpurascens*), but an antifungal effect has been reported that may inhibit the growth of other toxigenic phytopathogens [26].

This study takes a different approach than the analytical detection of mycotoxins. Based on the information about the spectrum of fungal infestation, it should be possible to make an early prediction about which mycotoxins might be present as part of quality assurance. 

In this context, Wawrzyniak made an interesting approach by predicting the level of fungal contamination of stored barley grain with the use of mathematical models based on the criterion of the early stage of mold development [27,28]. However, to date, for mycological routine analyses of barley kernels, the agar plating technique in combination with microscopic visualization is being used as the standard method in recent studies [29,30,31,32,33]. It must be taken into consideration that it often results in an incomplete picture of the microbial diversity: prevalent fungal species on the agar plate may become minorities in natural samples and vice versa. The method is laborious, time-consuming and well-trained personnel are a basic requirement. Visual assessment of malting barley is a quality assurance procedure for mycotoxin risk assessment at goods receipt in the brewing and malting industry. Nevertheless, the early stage of the infection is asymptomatic, although food safety concerns may already exist [16]. Therefore, there is a strong need for a culture-independent molecular method that provides fast screening of the (latent) toxigenic fungal spectrum. A technique such as the quantitative real-time polymerase chain reaction (qPCR), which amplifies a specific DNA sequence in the fungal genome, is a rapid, sensitive and specific alternative to these conventional detection methods to assess fungal infestation.

Several PCR approaches have been reported to detect seed-borne pathogens using the internal transcribed spacer (ITS) as standard marker for fungal DNA barcoding [33,34,35], non-transcribed spacer (NTS) or intergenic spacer regions (IGS) [36,37], mitochondrial DNA (mtDNA) [38], protein-coding genes or genes involved in the synthesis of mycotoxins. Quantitative real-time PCR assays based on SYBR green technology or fluorescent-labeled Taq-Man probes have shown high sensitivity for the detection and quantification of present fungal contamination in grains. For example, quantitative real-time PCR has been used to detect and quantify *Fusarium* spp. in cereals [39,40]; *Alternaria*, *Cladosporium*, *Fusarium* and *Penicillium verrucosum* in barley, rye and wheat [41]; the presence of mycotoxin-producing fungi in cereals and soybean for pig diets [42]; and *Alternaria* in cruciferous seeds [43]. Multiplex real-time PCR technique has been used by Vegi and Wolf-Hall [44] for the simultaneous detection and quantification of *Fusarium*, *Penicillium* and *Aspergillus* species in barley grains.

However, until now, only a limited amount of data regarding the contamination of naturally infected samples of malting barley raw material and the corresponding malt (*Hordeum vulgare* L.) before and after the malt house can be found in the literature. Within the broad range of filamentous fungi, the plant-pathogenic fungal genus *Fusarium* has been well-studied in this respect [16], while little attention has been paid to the members of the family Dematiaceae in previous studies, which has led this study to focus on these dark-pigmented fungi with further focus on potent mycotoxin-producing storage fungi being associated with dark kernel discoloration. 

The aim of the present study was to establish a quantitative real-time PCR assay based on SYBR green technology for detecting and quantifying DNA sequences of dark-pigmented fungal species on malting barley (*Hordeum vulgare* L.). To screen specifically for this required fungal spectrum, published oligonucleotides had to be validated for the fungal targets, and the assay was adapted to the barley matrix. The research conducted on filamentous fungi focused on dematiaceous fungi and dark-pigmented storage fungi, considered to be potent mycotoxin producers; in addition to *Fusarium*, they also play an important role in quality control (e.g., *Alternaria alternata*, *Cladosporium* spp., *Penicillium verrucosum*, *Aspergillus niger* and *Epicoccum nigrum*). The dataset was based both on industrial samples of different barley varieties and systematically on one variety across different proveniences in Germany accompanied by different climatic conditions (exposed to natural weather conditions) over four harvests. Using the developed qPCR method, the data were developed and compared with the conventional agar plate technique and a visual assessment to evaluate the suitability of these methods so batches possibly heavily contaminated with toxigenic fungi could be identified.

## 2. Materials and Methods

### 2.1. Grain Material

Naturally infected malting barley raw material (*Hordeum vulgare* L.) and the corresponding malt samples were obtained from the 2018, 2019, 2020 and 2021 harvests. Different climatic conditions were represented by one barley cultivar (Avalon, Saatzucht Breun, Herzogenaurach, Germany) grown on six different proveniences in Germany [45], in order to obtain a representative overview of the crop year and to balance regional outliers. A total of 48 barley samples were analyzed before and after malting by the qPCR assay and the plate method in three replications after homogenization and obtaining the representative samples.

Six different two-row spring malting barley varieties (Avalon, RGT Planet, Quench, Grace, Marthe and Laureate—malted industrial samples) from the 2017 harvest year were used to validate the specificity of the qPCR assay.

An additional 35 practical malting barley samples and the corresponding malts (1 kg micro malting plant according to the Mitteleuropäische Brautechnische Analysenkommission (MEBAK) method) were analyzed to elucidate the relationship between black kernel discoloration and fungal DNA content. The visual assessment of malting barley is a quality assurance procedure at goods receipt in the brewing and malting industry. To record the visible symptomatology, a representative sample of 200 g was taken in triplicate from each practice sample after homogenization (sample divider) and visually assessed according to the MEBAK raw material guidelines (R-200.02.701 2016-03). Barley quality was evaluated by determining the percentage of damaged barley kernels and the number of black- and red-discolored kernels. A total of 35 malt samples from the 2017–2020 harvest years were first optically evaluated, followed by DNA extraction and qPCR. The samples were selected based on their specificity with respect to the content of relevant black kernels: 0–3 (*n* = 12), 5–18 (*n* = 12) and 20–200 (*n* = 11). 

### 2.2. Malting Procedure

The micro malting procedure (1 kg) was performed following the standardized malting procedure according to the MEBAK raw material guidelines (R-110.00.008 2016-03). All the malting procedures were performed at the pilot-scale plant of the Chair of Brewing and Beverage Technology at the Technical University of Munich, Germany.

### 2.3. Fungal Strains

Fungal reference strains used for standard curves and positive controls were obtained from the DSMZ-German Collection of Microorganisms and Cell Cultures GmbH, Brunswick, Germany. The fungal cultures were cultivated under the recommended conditions specified for the strain and under a cycle of 12 h UV light and 12 h darkness. The fungal strains comprised three *Alternaria* species (*Alternaria alternata* DSMZ 62006, *A. solani* DSMZ 62028, *A. tenuissima* DSMZ 63360), *Epicoccum nigrum* DSMZ 11624, *Cladosporium cladosporioides* DSMZ 19653 and *Penicillium verrucosum* DSMZ 12639). With the exception of *Penicillium* sp., all of them are representatives of the form family Dematiaceae. For the quantification of the *Aspergillus niger* biomass, already isolated DNA was purchased due to the categorization into risk group 2 of the Biological Agents Ordinance.

### 2.4. Fungal DNA Extraction

Total genomic DNA of the fungal isolates was required to generate DNA standards for the quantitative polymerase chain reaction (qPCR). The method used here is a slightly modified version of the protocol of Tomita et al. [46]. In short, fungal mycelium (1 cm^2^) of a five- to eight-day-old culture was scrapped off the agar using a sterile slide and homogenized with 100 mg sterile 0.5 mm sand glass globules (Carl Roth, Karlsruhe, Germany). The ground material was mixed with 400 μL lysis buffer (50 mM Tris-HCl) (VWR, Radnor, PA, USA) pH 7.2, 50 mM EDTA (Merck, Darmstadt, Germany), 3% (*w*/*v*) SDS (AppliChem, Darmstadt, Germany), 1% β-mercaptoethanol (SIGMA-ALDRICH, St. Louis, MO, USA) and incubated at 65 °C for 60 min. A total of 200 μL phenol (Thermo Fisher Scientific, Waltham, MA, USA) was added before an incubation at 65 °C for 5 min. In total, 200 μL 24:1 chloroform (Th. Geyer, Renningen, Germany) to isoamyl alcohol (SIGMA-ALDRICH, St. Louis, MO, USA) was added, vortexed and then centrifuged (15 min, RT, 12,000 rpm). Further procedures were followed according to the author’s instructions. DNA quality was determined with the help of NanoDrop™2000c (Thermo Fisher Scientific, Waltham, MA, USA).

### 2.5. DNA Extraction from the Barley Samples

Genomic DNA from the barley grain was extracted according to the CTAB-based DNA extraction protocol recommended by the European Community Reference Laboratories for the isolation of maize DNA (European Commission CRLVL04/05XP) [47] with minor modification published by [48]. In brief, three biological replicas were prepared after homogenizing the samples and obtaining a representative sample. Barley grain and barley malt kernels were initially ground using a Tissue Lyser II bead mill (Qiagen, Düsseldorf, Germany). A total of 2 g of fine grain powder was lysed and precipitated with the respective cetyltrimetylammoniumbromide (CTAB) buffer, followed by chloroform-isoamyl extraction. DNA was precipitated from the aqueous phase and the pellet was washed twice in 70% ethanol, air dried and dissolved in 150 μL double-distilled water. The samples were stored overnight at 4 °C. The next day, the samples were centrifuged for 10 min at 13,000 rpm and 4 °C and the supernatant was transferred into a new reaction tube. The amount of DNA was determined using NanoDrop™2000c UV-Vis Spectrophotometer and the final DNA was adjusted to 100 ng/µL.

### 2.6. Agar-Plating Method

In order to ensure comparability to the molecular method, the experiment was carried out without surface sterilization of the seeds. In total, 100 randomly selected barley and barley malt kernels from each grain sample were placed on Petri dishes containing half-strength potato dextrose agar (PDA) (Merck, Darmstadt, Germany), chloramphenicol (100 mg/L) (Merck, Darmstadt, Germany) and Triton X-100 (0.1% (*v*/*v*)) (Merck, Darmstadt, Germany). Next, 5 seeds per Petri dish in 20 repetitions were incubated in a 12/12 h UV light (365 nm)/dark cycle at room temperature (20–24 °C), to promote sporulation. The fungal colonies were identified on days 7, 10 and 14 based on the taxonomic classification of the micro- and macroscopic features. For an exact determination, the mycelium of the laid-out sample material was transferred sterilely to a new selective medium until pure fungal isolates were generated.

### 2.7. qPCR Primers

Oligonucleotides for the detection of filamentous fungi commonly found in barley were synthesized by TIB MOLBIOL Syntheselabor GmbH, Berlin, Germany. In addition to primers specific to black fungi, a barley internal positive control, specific to the plant EF1α gene, was included for normalization, according to Nicolaisen et al. [39]. The primer sequences are shown in Table 1. Each primer pair’s specificity was evaluated against non-target DNA from barley grown in a sterile greenhouse. 

### 2.8. Quantitative Real-Time PCR Amplification

Absolute quantification of mycotoxin-producing fungi and barley DNA was performed by external standard calibration. Therefore, standard curves were generated using 1:10 serial dilutions of pure fungal and barley DNA. 

DNA amplification was performed in a LightCycler 480ll (Roche, Basel, Switzerland) using SYBR Green DNA intercalating dye. The samples were analyzed in three technical replicates, a no template control NTC (double-distilled water) was included in each run to check for external contamination and pure fungal DNA was used as positive control. Species-specific quantitative real-time PCR assays were carried out in a final reaction volume of 12 μL, containing 1X GreenMasterMix (Genaxxon bioscience, Ulm, Germany), 0.1 µM of each primer and 100 ng genomic DNA from barley samples. The assay consists of an initial denaturation step at 95 °C for 15 min, followed by 40 cycles of 95 °C for 20 s and 60 °C/65 °C for 60 s. A melting curve analysis was performed according to instrument instructions at 60 to 95 °C. The SYBR-specific fluorophore was quantified during the reaction by the instrument. The experimental analysis was conducted using the software LightCycler 480 SW 1.5 (Roche, Basel, Switzerland). The amount of individual black fungal DNA and barley DNA was calculated from C_q_ values using the external standard curves. The mean values from three biological replicas per sample were used. The grain infection rates were calculated as pg fungal DNA per ng plant DNA according to Nicolaisen et al. [39]. Statistical significance for the effects of the origin of the samples and the harvest year (weather conditions) on the fungal infection rate of the grain was determined by analysis of variance (ANOVA). The relationship between black grain discoloration and the amount of fungal DNA in barley malt was tested with the Pearson correlation coefficient. Both statistical analyses were performed using OriginLab 2018b. 

## 3. Results and Discussion

### 3.1. Mycological Status—Agar Plate Method

In order to determine the mycological status of naturally infected barley for brewing grown in Germany, a total of 4800 kernels (48 samples) of barley and the corresponding barley malt were mycologically evaluated using the agar plate method. The fungal cultures were identified and characterized by morphological criteria; the relative frequency of occurrence of the fungal genera was calculated per harvest year (Table 2) and for the growing regions (Table 3) to highlight the respective influencing factors.

**Figure 1:**
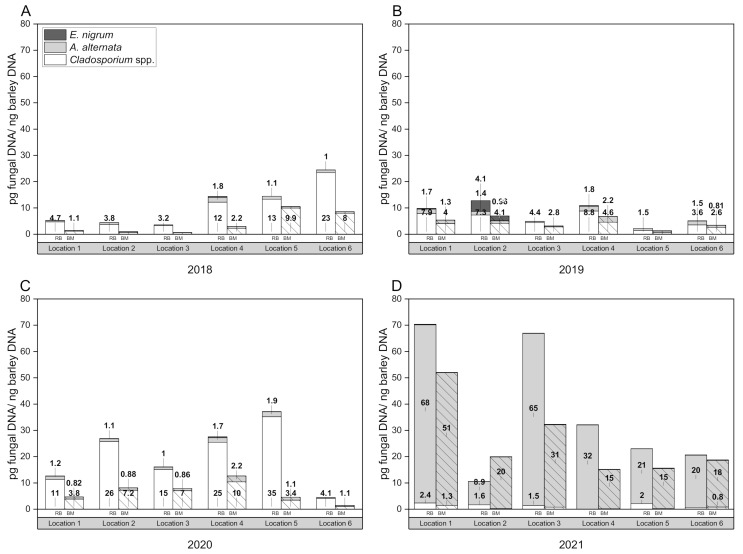
Amount of fungal DNA (pg fungal DNA per ng barley DNA) in the naturally infected barley raw material and in the corresponding malt grown in Germany. Fungal biomass was screened over four harvest years (2018–2021) using quantitative real-time PCR. The data represent mean values of six different proveniences. (**A**) 2018, (**B**) 2019, (**C**) 2020, (**D**) 2021.

Ubiquitous filamentous fungi grew out of almost all kernels. The spectrum of identified species belongs to genera of *Fusarium, Alternaria*, *Aspergillus*, *Penicillium*, *Rhizopus*, *Mucor*, *Cladosporium*, *Ulocladium*, *Bipolaris* and *Epicoccum* (Table 2 and Table 3). Figure 1 represents the composition of the mycoflora over four harvest years (2018–2021), separated into raw barley (24 samples) and the corresponding malt (24 samples). 

The results showed that high incidences of *Fusarium* species were detected in 2020 (58.1% in barley, 58.7% in malt) and 2021 (50.5% in barley and 77.7% in malt), while in 2018 *Fusarium* spp. were detected with a frequency of 2.2% in the barley and 24.6% in the malt. In the 2019 harvest year, *Fusarium* spp. were present with 29.9% in the raw barley and 27.7% in the malt. Species belonging to the genus *Alternaria* were the most frequently detected in the crop years of 2018 (36% in the raw barley and 31% in the malt) and 2019 (61% in the raw barley and 53% in the malt), while infestations declined sharply in 2020 with only 7% in the raw material and 14% in the malt; in 2021, the rate of *Alternaria* colonies in the raw fruit was 33%, and the rate in the malt declined to 9%. 

*Cladosporium* species were isolated from barley kernels at a frequency of 0.4% and with 8.5% from barley malt in 2018. During 2019, none of the fungal cultures of the barley belonged to the genus *Cladosporium,* whereas it was 7% on the malt kernels. In 2020, it was 1.7% on the barley and 10% on the malt. In 2021, higher values were found on the barley (9.4%) than on the malt (2.5%).

In 2018, *Epicoccum nigrum* colonies were identified with a low incidence of 0.4% in the raw barley and 0.8% in the malt. In 2019, there were 0.5% *E. nigrum* colonies on the barley and none on the corresponding malts. The opposite was the case in 2020: no growth was detected in the raw barley and 10.0% was detected in the barley malt. In 2021, 0.7% *E. nigrum* was found in the barley and 0.3% in the malt. In 2018, the genera *Penicillium* and *Rhizopus* were strongly represented (*Penicillium*: 29% barley, 16% malt; *Rhizopus*: 22% in barley, 18% in malt). The genus *Rhizopus* grew fastest at RT compared to other fungi, spreading strongly on the agar plate, and was therefore the dominant species among competing fungi; also, some *Fusarium* species did so.

*Alternaria* and *Fusarium* were the two most frequent fungal genera in malting barley. This is consistent with other studies that have reported on the barley malt mycoflora [30,54]. Moreover, higher incidences of *Alternaria* spp. colonies than *Fusarium* spp. during the 2018 and 2019 harvests are in line with previous studies [55,56,57]. Our findings about the mycoflora spectrum of malting barley and the corresponding malt in Germany are similar to those reported by other authors regarding barley cultivars in the Czech Republic [58], Slovak Republic [57], Romania [32], Lithuania [18] and Africa [59] (with the application of traditional microbiological methods).

Table 3 presents the summary of the mycoflora of the respective 6 proveniences pooled over 4 harvest years. The distribution of the distinct fungi varied with the geographic origin of the samples and the prevailing climatic conditions. Location 6 showed the highest level of infected grains with 65.0% *Fusarium* spp. in the barley and 70.2% in the malt. Location 4 was also dominated by *Fusarium* spp. (51.3% in RB, 62.8% in BM). In contrast, there was an increased occurrence of *Alternaria* spp. at locations 1 and 3 (44.5% and 37.9%; 47.0% and 27.2%). Storage fungi like *Penicillium* spp. and *Aspergillus* spp. were predominantly present in the barley, rather than in the barley malt; suggesting that the species are mainly surface contaminants of barley kernels. In the comparison between barley and the corresponding malt, fungi of the genus *Cladosporium* could mainly be detected in the malt using the culture technique, but not in the barley raw material. This may be due to the fact that their growth on the agar plate is suppressed by other faster-growing dominant fungi; the other theory would be that the fungus proliferates strongly during the malting process and can therefore be mainly detected in the malt. At this point, one limitation of the culture-based method becomes apparent: in some growing regions, the abundance of fast-growing fungi could have affected the detection of other fungal colonies, thereby falsifying the result.

### 3.2. qPCR Assay Adaption

In addition to the agar plate method, a quantitative real-time PCR analysis was conducted to determine the exact amount of selected fungal DNA in barley and barley malt samples—especially to target those fungi that are difficult to cultivate on the agar plate and to disregard the effect of overgrowth and competition. This study specifically targets the most abundant dark-pigmented filamentous fungi which are considered hazardous to human health and are related to cereals.

Since the oligonucleotides used in this work were previously published [39,49,50,51,52,53], the assay had to be adapted to the barley matrix. All fungal primers were checked in silico for cross-hybridization with other fungal genera, yeasts and bacteria with the Basic Local Alignment Search Tool (BLAST) [60] prior to ordering (data not shown). Each fungal primer pair´s specificity was evaluated against non-target DNA from sterile greenhouse grown barley and DNA extracted from pure fungal cultures. In the SYBR real-time PCR assays, all target fungi isolates gave strong positive fluorescent signals, while the non-targeted fungal strains produced no signals. It is the case that 60 °C has proven to be the optimum annealing temperature for Hor1F/Hor1R and PVerFor/PVerRev; 65 °C was used for AaltFor/AaltRev, CladoFor/CladoRev, ENiFor/ENiRev and AspNiF/AspNiR.

A melting curve analysis to determine the reaction specificity was performed immediately following the respective real-time PCR assay. All reactions showed one single peak indicating a specific amplification of the target DNA sequences and the absence of cross-reaction with other fungal species or the formation of primer-dimer artifacts. To investigate whether the barley matrix had an inhibitory effect on the real-time PCR performance, spiking experiments were performed in which DNA extracted from the barley source was spiked with pure fungal DNA. No changes in the cp values could be observed, therefore primers were considered to be highly specific for the quantification of fungal DNA in barley and malt.

Regarding the EF1α assay, all barley variety isolates showed C_q_ values between 19.85 and 22.04, which means that the samples are highly comparable with each other and show good extraction efficiency. In the post-amplification melting curve analysis, all six tested malting barley varieties (Avalon, Planet, Quench, Grace, Marthe and Laureate) produced a single distinct peak, representing a pure, single amplicon. NTC and fungal DNA showed no amplification curves, as expected. According to this, the primer pair EF1α binds exclusively to genomic DNA of *Hordeum vulgare*, without any cross-amplification between the barley variations. The sterile barley reference was used to validate further fungal primer pairs.

The efficiency of the respective primer pairs was tested by standard curves generated using serial dilutions of pure fungal DNA. C_q_ values were plotted against the known template concentrations, resulting in slope values between 3.228 and 3.544 (Hor1F/Hor1R: 3.228, AaltFor/AaltRev: 3.380; CladoFor/CladoRev: 3.393; PVerFor/PVerRev: 3.351, ENiFor/ENiRev: 3.544, AspNiF/AspNiR: 3.318). Efficiencies ranged between 1.915 and 2.041 (Hor1F/Hor1R: 2.041 (104.1%), AaltFor/AaltRev: 1.976 (97.6%); CladoFor/CladoRev: 1.971 (97.1%); PVerFor/PVerRev: 1.988 (98.8%); ENiFor/ENiRev: 1.915 (91.5%); AspNiF/AspNiR: 2.002 (100.2%). Therefore, the requirements are considered acceptable according to Bustin et al. [61]. The detection limit of the respective assays ranged from 100 ng fungal DNA to 0.001 ng for *Alternaria alternata*; from 100 ng to 0.01 ng for *Hordeum vulgare*, *Cladosporium* spp., *Penicillium verrucosum* and *Aspergillus niger*; and from 100 ng to 0.0001 ng for *Epicoccum nigrum*. 

### 3.3. Mycological Status—Quantification of Fungal DNA

The use of external standards enables the quantification of fungal DNA. Infection levels of barley raw material and barley malt samples were calculated from the three biological replicates, amplificated in triplicate. Fungal DNA concentrations were normalized for analysis with plant control EF1α concentrations of the same sample. A total of 48 barley samples were analyzed using the qPCR assays for *A. alternata*, *Cladosporium* spp., *Epicoccum nigrum*, *P. verrucosum* and *A. niger*. 

The results of the molecular analysis are shown graphically in Figure 1 as mean values of infection levels (pg fungal DNA per ng barley DNA). The assays were able to detect and quantify *A. alternata* and *Cladosporium* spp. DNA in all examined barley samples. The data obtained in this study result in the cumulative total DNA contents presented in Table 4. The molecular analysis revealed that DNA of the highly toxigenic storage fungi *P. verrucosum* and *A. niger* was not detectable throughout the examined samples. However, the preliminary tests confirmed the specificity of the primers and the spiking experiments were also positive. Therefore, the cultures identified on the agar plates as *Penicillium* spp. and *Aspergillus* spp. did not include the strong toxin-producing ones we were looking for in quality control.

The variability in fungal incidence levels between locations and harvest years is thought to be due to variance in environmental conditions. However, statistical analysis has shown that the provenance has no significant effect on the fungal infestation level (pg fungal DNA per ng barley DNA) in the case of *A. alternata* (F = 0.95, *p* = 0.46) and *Cladosporium* spp. (F = 0.99, *p* = 0.43), but the year of harvest was found to be significant (*A. alternata*: F = 20.92, *p* = 1.04 × 10^−9^; *Cladosporium* spp.: F = 10.65, *p* = 7.93 × 10^−6^). With regard to the fungus *Epicoccum nigrum*, it was proven that both the provenance (F = 3.06, *p* = 0.02) and the year of harvest (F = 3.09, *p* = 0.03) have a significant influence on the amount of fungal DNA on the grain. These results are consistent with the harvest reports for spring barley in Germany: during 2018 and 2019, dry and warm weather conditions prevailed in June and July; hot and rain-free ripening resulted in healthy barley plants and a visually flawless harvest [62,63]. This is also reflected in the low amounts of *A. alternata* DNA in 2018, with higher values at individual locations (4, 5 and 6), which was consistent with *Cladosporium* spp. DNA. During 2019, DNA levels were almost equal in all growing regions, but were slightly higher than in 2018. The 2020 crop year had good water supply and moderate temperatures during the tillering and grain-filling phase. After a warm, mostly rain-free ripening, there was persistent rainfall during the harvest [64]. The DNA content of *A. alternata* remained quite similar as in the previous year, while the *Cladosporium* spp. DNA values were very high in 2020. Location 6 was the only provenience that had a similar infection level as the previous year. High incidences in 2021, especially at location 1 and 3, could be due to moist weather conditions, waterlogging and lack of sunlight. Weather-related harvest delays resulted in long standing times, increased water contents and reduced optical quality [65], what seemed to have provided the most favorable conditions for *A. alternata*. This is consistent with previous studies that confirmed a relationship between disease incidence level and agro-environmental factors, such as crop year and microclimatic conditions [55,57,66,67]. Heavy precipitation during the early stage of grain development (milk and soft dough stage) has been related to an increased incidence of black point [68]. To further investigate this aspect using the data obtained here, a mathematical approach in which the infection level is expressed as a function of an independent variable describing the atmospheric conditions would be interesting. This could be a promising follow-up for further research. However, the fungal DNA concentrations obtained in this study are consistent with comparable studies. Thus, similar DNA contents were detected by Kulik et al. (2014) in organic grains and a positive correlation between *Alternaria* and *Cladosporium* was found [41]. Okorski et al. detected *P. verrucosum* DNA in pig diets within the range of 0.7 pg to 2.1 pg [42]. Regarding *Fusarium* spp. DNA, Nicolaisen et al. found 0–26.2 pg/ng, depending on the respective species in wheat samples. Furthermore, a relationship between DON content and the amount of *F. graminearum* and *F. culmorum* DNA was shown [39]. A positive correlation between the PCR detection of fungal DNA and mycotoxins has been demonstrated for *Alternaria* DNA and the presence of ALT, AOH or AME in raw and processed tomato products [69]. Moreover, Orina et al. reported a correlation between *Alternaria* DNA concentration and TeA content in grain samples from the Urals and West Siberia [70]. However, Schmidt-Heydt et al. showed that qPCR data for the presence of *P. verrucosum* in wheat do not necessarily correlate with the synthesis of ochratoxin A, as the fungus requires a certain adaptation time [52]. The extent to which the partly very high fungal DNA contents detected in this study are associated with increased mycotoxin levels must be clarified in further investigations.

In summary, the results obtained demonstrate that the quantitative real-time PCR is a valid method to detect the presence of toxin-producing fungi in barley raw material and barley malt samples. By using species-specific primers, it is possible to screen specifically for the dark-pigmented fungi *A. alternata*, *Cladosporium* spp. and *E. nigrum*. Infection levels of samples can be calculated exactly; even small DNA amounts can be detected. Precisely for this reason, qPCR could be useful for the early detection of potential risks of mycotoxin contamination in barley and barley malt batches.

### 3.4. Mycological Status—Comparison between the Agar Plate Method and qPCR

The DNA-based method allowed accurate detection and quantification of the fungal biomass of individual species relevant in brewing cereals. It could be demonstrated that the DNA of *A. alternata* and *Cladosporium* spp. was present in all examined barley samples, even when the agar cultivation could not determine the presence of these fungi.

Using molecular biological analysis, *A. alternata* DNA was detected in all barley and malt samples examined, even in samples that did not show visible *Alternaria* growth on the agar plate (individual locations, not shown). On the contrary, these locations actually had the highest *A. alternata* load according to the molecular method. *Cladosporium* spp. DNA was also present in large amounts in all samples tested, both in barley and in malt. On the agar plate, *Cladosporium* was detected mainly in the malt, but only sporadically in the barley. Accordingly, real-time PCR makes it possible to detect fungal contamination that has not grown on the agar plate. DNA from *Aspergillus niger* and *Penicillium verrucosum*, both strong mycotoxin producers, was not detectable in any of the samples tested, although many representatives of the genus *Penicillium* were identified on the agar plate, particularly in the 2018 crop year. This makes qPCR an additional tool for identifying lots potentially contaminated with toxigenic fungi. It allows unambiguous identification of fungi, which based on lower level (species) morphology alone, is not always clear, especially for untrained personnel.

The comparison of both methods revealed only limited consensus. The traditional agar cultivation method provides an initial overview of the total fungal community, in percentage of infected kernels, with few resources. However, identification by morphological criteria alone does not reliably cover all species, due to preselection through culture media and incubation conditions. One problem is that growth rates between fungal strains are not the same; dominant, fast-growing or strongly spreading strains, such as *Rhizopus* spp., *Fusarium* spp., *Penicillium* spp. and *Aspergillus* spp. may overgrow plates; additionally, not all species can be cultivated in media. Furthermore, this method is laborious, time-consuming and requires qualified personnel with extensive knowledge of fungal taxonomy for lower-level species identification.

In contrast, culture-independent molecular methods, such as quantitative real-time PCR, can detect any individual target species, regardless of the growing conditions, even non-culturable pathogens. Main advantages include high sensitivity and specificity. This technique allows an accurate and efficient quantification of fungal DNA, which enables precise assessment of the level of infection and comparability between samples. Furthermore, it offers rapid and simultaneous testing of a large number of samples, suitable for barley raw material and malt.

In summary, the two methods provide different types of information: the culture-based method is suitable for a first overview, even if not all species can be reliably detected. It does not provide information about the total level of infestation (quantitative). Accurate detection of selected toxigenic fungi can be obtained using quantitative real-time PCR assays. It enables obtaining information on the fungal infestation of cereals within a short period of time. Based on this work and adapted to additional species, qPCR can be used in the future to ensure the safety and quality of raw materials.

### 3.5. Mycological Status—Before and after the Malt House 

In summary, the investigation of the infection level over four harvest years analyzed by qPCR showed that the barley raw material is more highly contaminated than the corresponding malt. On average, the fungal biomass was reduced after malting by 58.57% in the case of *A. alternata*, by 28.27% for *Cladosporium* spp. and by 12.79% for *Epicoccum nigrum*. Normalization with the pure barley DNA considers the extraction efficiency and can therefore be excluded as a possible source of interference. The higher load in the raw material can be explained by surface pathogens, which are removed during the malting process (intensive cleaning steps: mainly wet steeping, thermal load: kilning, deculming/malt cleaning). 

Without prior treatment, malting barley carries a high microbial load when entering the malting process [71,72]. During steeping, the increased water content initiates the germination of the grain; this is also the most critical step, as it could favor fungal growth and the activation of dormant spores [5,73]. However, the steeping itself can also reduce the fungal contamination of barley by washing off both dust and the fungal mycelium on the surface [74]. Although some studies have reported that fungal loads can increase by two to five times the original amount during germination [2,4,11,73,74,75], comparison studies have shown contradictory findings: the levels of field fungi such as *Alternaria* and *Cladosporium* declined during germination [73,76]. A reduction in fungal contamination after kilning or malt cleaning agrees with the results of Ackermann [59], Kocić-Tanackov et al. [77], Juste et al. [73] and Douglas and Flanningan [76], although these findings were based on the use of the traditional cultivation-based methods. Studies in which molecular analysis investigated black fungi contamination before and after the malting process are not yet available. The study showed that qPCR analysis allows more accurate quantification of fungal DNA and thus a comparison of these two steps with each other.

To the best of our knowledge, there is no literature on the behavior of *Epicoccum nigrum* before or after malting. This research confirmed that *E. nigrum* biomass is lower in malt than in barley. The same could be found regarding *A. alternata* and *Cladosporium* spp. 

For practical purposes (malt houses and breweries), this means that presumably a major amount of the initial black fungal load is removed throughout the malting process. This is an important basis for further research directions. It must be clarified how the kinetics of these fungi behave during germination—whether they grow strongly and are then removed during cleaning or whether they decrease continuously over the complete process. Additionally, the relationship to mycotoxin formation during the malting process needs to be clarified.

### 3.6. Relationship between Kernel Discoloration and Fungal Biomass

To finally investigate the relationship between the amount of fungal DNA in barley malt measured by quantitative real-time PCR and the number of black kernels in 200 g malt (visual assessment according to MEBAK R-200.02.701 (2016-03)), a Pearson correlation analysis was carried out for 35 individual malt samples collected in 2017–2021. The number of discolored black grains ranged between 0 and 200, divided into 3 subgroups: no symptomatology (0–2 black kernels; *n* = 10), low symptomatology (3–14 black kernels; *n* = 13) and high symptomatology (18–200 black kernels; *n* = 12). The DNA content of group 1 ranged between 9.02 pg/ng and 26.94 pg/ng for *A. alternata*, although in some instances there were no visible symptoms present at all. Group 2 ranged from 1.47 pg/ng to 40.72 pg/ng *A. alternata* DNA and group 3 from 6.79 pg/ng to 24.73 pg/ng. Regarding *Cladosporium* spp., group 1 included DNA amounts between 0.51 pg/ng and 4.52 pg/ng, group 2 DNA amounts between 0.71 pg/ng and 7.08 ng/pg and group 3 DNA amounts between 1.27 pg/ng and 6.12 pg/ng. *Epicoccum nigrum* DNA was detected in group 1, with an incidence between 0 pg/ng and 32.14 pg/ng; in group 2 DNA incidence was between 0.01 pg/ng and 86.26 pg/ng and in group 3 DNA incidence was between 0.02 pg/ng and 39.22 pg/ng. The DNA of *A. niger* and *P. verrucosum* could not be detected in these samples either.

**Figure 2:**
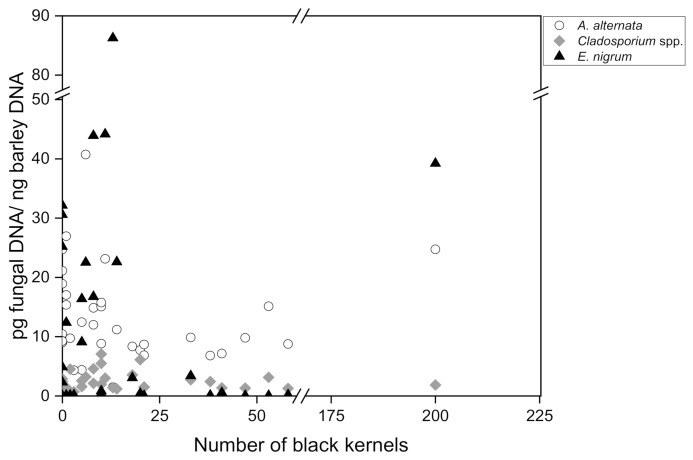
Correlation of black kernel symptomatology and the amount of fungal DNA. ○ = *A. alternata*, ◆ = *Cladosporium* spp., ▲ = *Epicoccum nigrum* in barley malts of the harvest years 2020 (*n* = 10), 2019 (*n* = 10) and 2017 (*n* = 15).

The amount of *A. alternata*, *Cladosporium* spp. and *E. nigrum* DNA was plotted against the number of black kernels (Figure 2). According to the statistics, no causal relationship was found for *A. alternata* (r = 0.105), *Cladosporium* spp. (r = −0.031) and *Epicoccum nigrum* (r = 0.118). Consequently, with regard to the black grain discolorations, no direct correlation with an increased occurrence of fungal DNA concentrations of the species *A. alternata*, *Cladosporium* spp., *Epicoccum nigrum*, *Penicillium verrucosum* and *Aspergillus niger* could be determined. Moreover, the absence of black grain discoloration in a malt batch is not a clear indication that fungal contamination is not present, since high amounts of fungal DNA were detectable even in samples that did not exhibit disease symptoms.

Black grain discoloration has been associated with the presence of dematiaceous fungi; *Alternaria* spp., *Cladosporium* spp., *Bipolaris* spp., *Drechslera* spp., *Chaetomium* spp. and *Epicoccum nigrum* have been isolated from symptomatic kernels [78,79,80,81]. Nevertheless, discoloration may also be due to other fungal genera such as *Aspergillus*, *Penicillium* and *Fusarium* [78,81]. A clear relationship between symptom intensity and infestation level has not yet been demonstrated; however, recent studies confirm a notable association with environmental stress [68,79,82,83]. Similar findings regarding the optical scoring for *Fusarium* spp. on malting barley were reported by Geißinger et al. The visual assessment of grain based on a defined number of red kernels was described as a useful tool, but was not decisive for risk assessment [16].

The results obtained in this study have practical relevance for (visual) quality assurance (during goods reception), as it was shown that the number of black kernels is not significant for the presence or absence of black toxigenic fungi. Consequently, mycotoxins may also be present in supposedly safe raw material and can pose a risk to food safety. Measuring fungal DNA provides a more valuable tool for identifying batches that are highly contaminated with toxigenic fungi. Nevertheless, further research is needed to clarify the relationship between fungal biomass (DNA), black grain discoloration and mycotoxin content, to give this method even more significance.

## 4. Conclusions

In the present study, a real-time PCR assay was adapted to detect and quantify ubiquitous dematiaceous fungal DNA in barley and corresponding barley malt samples. The results demonstrate that qPCR is a sensitive and specific method for quantifying the fungal biomass of individual species relevant in brewing cereals. The assays were able to detect and quantify *A. alternata* and *Cladosporium* species in all examined barley samples, even without visible mold contamination. 

In comparison with the culture-based method, it was shown by incubating grains on a PDA medium that the traditional method does not reliably cover all species due to preselection through culture media and incubation conditions. In summary, the two methods provide different types of information: the culture-based method is suitable for a first overview, as it may not always perform well for lower-level identification. Accurate detection of selected toxigenic fungi can be obtained using quantitative real-time PCR assays. It enables information on the fungal infestation of cereals within a short period of time. 

By examining different proveniences in four consecutive harvest years, fortunately including years with extreme weather conditions, a relationship between the extent of disease infestation levels and agro-environmental factors was confirmed. Humid weather conditions appeared to provide the most favorable conditions for fungal occurrence, while hot and rainless periods resulted in low incidence levels.

The investigation of the infection level (fungal DNA, analyzed by qPCR) showed that the barley raw material is more highly contaminated than the corresponding malt. The steps of wet steeping, kilning and deculming/malt cleaning presumably removed a major amount of the initial black fungal load through the malting process. On average, the fungal biomass was reduced after malting by 58.57% in the case of *A. alternata*, by 28.27% for *Cladosporium* spp. and 12.79% for *Epicoccum nigrum*. 

With regard to the relationship between black grain discolorations and infestation, no direct correlation to an increased occurrence of fungal DNA concentrations of the species *A. alternata*, *Cladosporium* spp., *Epicoccum nigrum*, *Penicillium verrucosum* and *Aspergillus niger* could be determined. In addition, the absence of black grain discoloration in a malt batch is not a clear indication that fungal contamination is not present, since high amounts of fungal DNA were detectable even in samples that did not exhibit disease symptoms. Regardless of whether symptoms are present or not, a cross-screening is recommended as part of quality assurance, since an infestation cannot be excluded on the basis of visual examination alone.

Measuring fungal DNA by qPCR provides a highly sensitive and time-saving screening of brewing grains for latent fungal infections in order to identify batches that are potentially highly contaminated with toxigenic fungi. If an increased fungal infestation is detected using this method, a further analysis of the batch is recommended for mycotoxins according to the MEBAK guidelines. Nevertheless, further research is needed to clarify the relationship between fungal biomass (DNA), black grain discoloration and mycotoxin content, in order to give this method even more significance.

## Figures and Tables

**Table 1:** Oligonucleotides used for the detection and quantification of the fungi of interest in barley and barley malt kernels via qPCR assay.

Target Species	Oligo Name	Sequence (5′–3′)	References
*Alternaria alternata*	AaltFor/AaltRev	GTGCCTTCCCCCAAGGTCTCCG CGGAAACGAGGTGGTTCAGGTC	Kordalewska et al., 2015 [49]
*Cladosporium cladosporioides*	CladoFor/CladoRev	TACTCCAATGGTTCTAATATTTTCCTCTC GGGTACCTAGACAGTATTTCTAGCCT	Zeng et al., 2005 [50]
*Epicoccum nigrum*	ENiFor/ENiRev	CCTAGAGTTTGTAGACTTCGGT GACGTCGTCGTTATGAGTG	Martini et al., 2009 [51]
*Penicillium verrucosum*	PVerFor/PVerRev	TTGCGAATCAGGGTCCAAGTA CGAGCATCGAAAGCAAAAACA	Schmidt-Heydt et al., 2009 [52]
*Aspergillus niger*	AspNiF/AspNiR	GGGCAAAGGGTTGGGTCTTC GACGAGGACGGCACGAGGA	Morgan von Hertwig et al., 2018 [53]
*Hordeum vulgare*	Hor1F/Hor1R	TCTCTGGGTTTGAGGGTGAC GGCCCTTGTACCAGTCAAGGT	Nicolaisen et al., 2009 [39]

**Table 2:** Relative abundance of the 9 dominant fungal genera isolated from the respective 100 kernels of raw barley (RB) and corresponding barley malt (BM) of the 2018–2021 harvest years; number of different samples = 48.

Year	Parameter	*Fusarium* spp.	*Alternaria* spp.	*Cladosporium* spp.	*Penicillium* spp.	*Aspergillus* spp.	*Epicoccum nigrum*	*Rhizopus* spp.	*Mucor* spp.	*Ulocladium* spp.	Others
2018	RB	0.022	0.357	0.004	0.286	0.098	0.004	0.219	0	0.004	0.004
	BM	0.246	0.305	0.085	0.157	0.008	0.008	0.182	0	0.004	0.004
2019	RB	0.299	0.612	0	0.010	0.010	0.005	0.065	0	0	0
	BM	0.277	0.533	0.070	0.014	0.014	0	0.035	0.028	0.028	0.003
2020	RB	0.581	0.066	0.017	0.156	0.123	0	0.050	0	0.007	0
	BM	0.587	0.135	0.100	0.023	0.010	0.010	0.092	0.010	0013	0.020
2021	RB	0.505	0.334	0.094	0.021	0.024	0.007	0.003	0	0.003	0.010
	BM	0.777	0.093	0.025	0.006	0.033	0.003	0.062	0	0	0

**Table 3:** Impact of the proveniences on the fungal spectrum: relative abundance of fungal genera isolated from kernels of raw barley (RB) and the corresponding barley malt (BM) of six proveniences in Germany summarized over 4 harvest years (2018–2021); standardized malting procedure according to MEBAK raw material (R-110.00.008 2016-03); number of different samples = 48; *n* = 400 per location and parameter (100 kernels per year).

Location	Parameter	*Fusarium* spp.	*Alternaria* spp.	*Cladosporium* spp.	*Penicillium* spp.	*Aspergillus* spp.	*Epicoccum nigrum*	*Rhizopus* spp.	*Mucor* spp.	*Ulocladium* spp.	Others
1	RB	0.161	0.445	0.088	0.088	0.204	0.007	0	0	0	0.007
	BM	0.280	0.379	0.056	0.201	0.019	0.005	0.374	0.005	0.014	0.005
2	RB	0.266	0.383	0.008	0.227	0.055	0.008	0.055	0	0	0
	BM	0.441	0.330	0.117	0.011	0	0.016	0.320	0.011	0.016	0.027
3	RB	0.313	0.470	0.006	0.006	0.048	0.006	0.084	0	0.006	0
	BM	0.497	0.272	0.139	0	0.026	0.007	0.003	0	0.020	0.007
4	RB	0.513	0.146	0.015	0.221	0.045	0	0.055	0	0.005	0
	BM	0.628	0.233	0.247	0.015	0.050	0	0.050	0	0	0
5	RB	0.285	0.388	0.085	0.091	0.050	0	0.091	0	0.012	0
	BM	0.411	0.265	0.060	0.011	0.005	0	0.184	0.043	0.022	0
6	RB	0.650	0.097	0.005	0.036	0.041	0.005	0.157	0	0	0.010
	BM	0.702	0.083	0.033	0	0.008	0.004	0.165	0	0	0.004

**Table 4:** Cumulative fungal DNA contents (pg fungal DNA per ng barley DNA) per harvest year (2018–2021) in raw barley (RB) and barley malt (BM); analyzed by quantitative real-time PCR; n.d. = not detected.

Year	Parameter	*Epicoccum* *nigrum*	*Alternaria alternata*	*Cladosporium* spp.	*Penicillium verrucosum*	*Aspergillus niger*
2018	RB	0.47	0.91	60.62	n.d.	n.d.
	BM	0.05	0.43	22.34	n.d.	n.d.
2019	RB	4.69	1.27	33.36	n.d.	n.d.
	BM	2.05	1.04	18.70	n.d.	n.d.
2020	RB	0.68	1.20	116.95	n.d.	n.d.
	BM	0.97	1.06	32.98	n.d.	n.d.
2021	RB	0.54	215.23	8.05	n.d.	n.d.
	BM	0.82	149.47	3.59	n.d.	n.d.

## Data Availability

The data presented in this study are available on request from the corresponding author.
